# Sex differences in impact of sarcopenia on falls in community-dwelling Korean older adults

**DOI:** 10.1186/s12877-021-02688-8

**Published:** 2021-12-18

**Authors:** Yunsoo Soh, Chang Won Won

**Affiliations:** 1 Department of Physical Medicine and Rehabilitation, Kyung Hee University Medical Center, 23 Kyungheedae-ro, Dongdaemoon-gu, Seoul, 02447 Republic of Korea; 2 Department of Family Medicine, Kyung Hee University Medical Center, 23 Kyungheedae-ro, Dongdaemoon-gu, Seoul, 02447 Republic of Korea

**Keywords:** Aging, Sarcopenia, Falls, Fall-related fractures, Asian working group for sarcopenia, Body composition

## Abstract

**Background:**

Falls are one of the most serious health problems among older adults. Sarcopenia is characterized by a decrease in muscle mass, strength, and physical function. Due to potentially age-related conditions, both falls and sarcopenia have common risk factors. However, the association between sarcopenia and falls is controversial. Moreover, the sex differences in the impact of sarcopenia on falls is not yet clear. This study aimed to investigate the sex differences in the impact of sarcopenia, defined by the Asian Working Group for Sarcopenia (AWGS), on falls in Korean older adults.

**Methods:**

In this cross-sectional study, we used data from the Korean Frailty and Aging Cohort Study; 2323 community-dwelling older adults (1111 males and 1212 females) aged 70–84 years were recruited in this cross-sectional study. To evaluate sarcopenia, the AWGS diagnostic algorithm was used. We compared the faller and non-faller groups. We performed unadjusted and fully adjusted logistic regression analyses to evaluate the relationship between sarcopenia, falls, and fall-related fractures.

**Results:**

A total of 239 (24.1%) females in the faller group had a history of falls in the past year, which was statistically higher than that in males (176, 15.8%). In the fully adjusted model, handgrip strength (odds ratio [OR] = 1.508, 95% confidence interval [CI] = 1.028–2.211), and short physical performance battery (OR = 2.068, 95% CI = 1.308–3.271) were significantly lower in the male faller group. However, in the fully adjusted model, the female faller group only showed a significantly low appendicular skeletal muscle mass index (OR = 1.419, 95% CI = 1.058–1.903).

**Conclusions:**

This large cohort study aimed to identify the sex differences in the incidence of sarcopenia in the older Korean population, using the AWGS diagnostic algorithm, and its correlation with falls and fall-related fractures. The incidence of falls did not increase in the sarcopenia group. Among the sarcopenia components, sex differences affect the history of falls. Therefore, when studying the risk of falls in old age, sex differences should be considered.

## Background

Falls are one of the most serious problems that threaten the health of older adults and untimely cause death, physical injury, immobility, socio-psychological dysfunction, and hospitalization [1]. Fall risk factors in older adults include aging, female sex, living alone, cognitive impairment, fear of falling, vitamin D deficiency, multiple medications, visual impairment, and chronic comorbidities such as cardiopulmonary disease, depression, osteoarthritis, and diabetes [2–5]. The risk of falls increases dramatically in people aged > 75 years. In the community-dwelling population, one-third of older adults aged > 65 years and 50% individuals aged > 80 years experience falls each year; among them, > 40% experience recurrence [6]. Approximately 20–30% of fallers sustain fall-related injuries that reduce their mobility and independence in performing the activities of daily living and increase the risk of premature death [7].

The physiological changes that usually occur due to aging vary, and one of them is sarcopenia, caused by a decrease in skeletal muscle mass. Sarcopenia directly causes a decrease in muscle strength and physical functions, thus causing disability and increasing the risk of mortality [8, 9]. Muscle mass and strength decrease as a person ages, and changes in body composition occur in both males and females; these changes occur regardless of the changes in body weight [10]. The prevalence of sarcopenia differs depending on the diagnostic method used and sex; sarcopenia occurs in 10% individuals aged > 65 years, and its prevalence increases with age by > 50% after the age of 80 years [11–13].

The factors related to the development of sarcopenia include reduction in muscle cells; imbalance between protein breakdown and synthesis; decrease in the levels of inflammatory cytokines, cortisol, and sex hormones; insulin resistance; and lifestyle-related factors such as nutritional intake and physical activity. In particular, lifestyle-related factors are the primary risk factors for sarcopenia, including malnutrition, reduced physical activity, alcohol intake, and smoking [14]. With aging, the skeletal muscle mass, muscle strength, endurance, and contractility are affected, and an individual’s sense of balance decreases. Moreover, this decrease in proprioceptive sensory function leads to a decrease in the ability to balance, which causes difficulties in motor control. Changes in these factors lead to a decrease in physical function and slowness in gait, thus increasing the risk of falls in older adults [15]. According to a recent Asian study, there is a difference in the prevalence of sarcopenia between sexes (19.2% in males and 8.6% in females), and sex differences may affect fall risk, but this has not yet been studied [16]. Moreover, obesity or sarcopenic obesity, another risk factor for falls, is more common in elderly females [17]. Therefore, sex differences may affect the risk of falls in patients with sarcopenia.

This study aimed to investigate the sex differences in the impact of sarcopenia, defined by the AWGS, on falls in community-dwelling Korean older adults using baseline data from the Korean Frailty and Aging Cohort Study (KFACS).

## Methods

### Data and study population

**Fig. 1:**
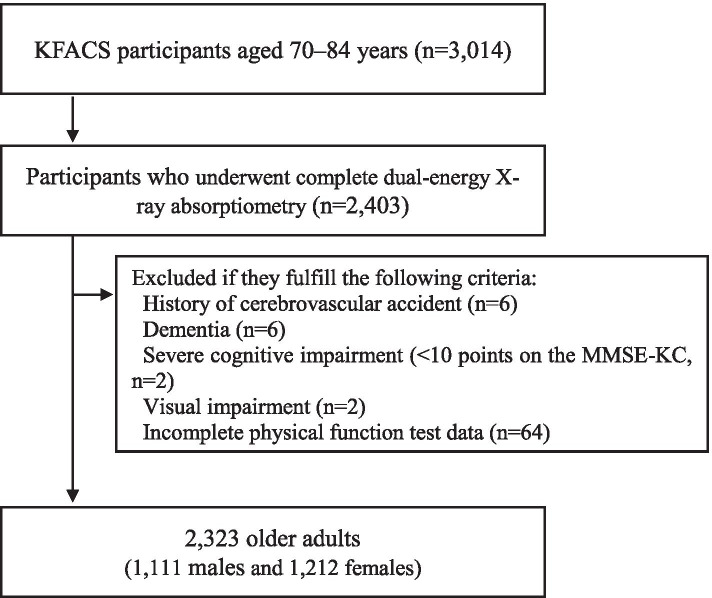
Flow chart of the participant recruitment process. KFACS, Korean Frailty and Aging Cohort Study; MMSE-KC, Mini-Mental Status Examination in the Korean version of the CERAD assessment packet

In this cross-sectional study, we used the data from the 2016–2017 KFACS to investigate the relationship between sarcopenia, defined by the AWGS, and falls. The KFACS is a nationwide, multicenter study performed in eight medical and two public health centers across South Korea in community-dwelling older adults aged 70–84 years. A total of 3014 participants with incomplete data on weakness due to cerebrovascular accident, with a deformity or motor deficit in the extremities, with severe cognitive impairment and blindness, with inability to complete the short physical performance battery (SPPB) test, and without DEXA data were excluded. Finally, 2323 participants (1111 males and 1212 females) were included (Fig. 1). Data on baseline demographics and medical history, including age, sex, years of education (< 6 years, 6–12 years, or > 12 years), location of residence (rural or city), smoking status, alcohol consumption, body mass index (BMI), and chronic comorbidities, were also collected. Participants who smoked more than one cigarette per week and drank alcohol at least once a week were defined as smokers and drinkers, respectively. The KFACS protocol was approved by the Institutional Review Board (IRB) of the Clinical Research Ethics Committee of Kyung Hee University Medical Center (IRB number: 2015–12-103), and all participants provided written informed consent.

### Sarcopenia

Sarcopenia was defined using the AWGS diagnostic criteria, which was updated in 2019 [18]. Participants with a low muscle mass and either low muscle strength or low physical performance were characterized as having sarcopenia. Participants characterized as having sarcopenia with a low muscle mass, and both low muscle strength and low physical performance were further characterized as having severe sarcopenia.

1. Muscle mass: DEXA is more widely used as it is a noninvasive, accurate, and convenient method for measuring muscle quantity [19]. Using DEXA, we calculated the appendicular skeletal muscle mass index (ASMI) at different heights, and the results are expressed as ASMI/height^2^ (cutoff values: < 7.0 kg/m^2^ for males and < 5.4 kg/m^2^ for females).
2. Muscle strength: Using a hand dynamometer (JAMAR, Bolingbrook, IL, USA), the participants’ handgrip strength (HGS) on both sides was measured twice, with the elbow flexed at 90° while in a sitting position, and the highest value was obtained (cutoff values: < 28 kg for males and < 18 kg for females).
3. Physical performance: The SPPB test is a well-established physical performance test that evaluates the lower extremities in older adults. This test assesses the following items: standing balance, 4-m walking speed, and five repeated chair stands. Each item was scored 4 points, with a higher score indicating better lower extremity physical function. In the AWGS diagnostic criteria, a score of ≤9 points indicates low physical performance.

### Falls and fall-related fracture

Falls was confirmed based on the participants’ response to the following question: In the past year, have you had falls? “Falls” was defined as a sudden contact of any part of the body, except the feet, with the ground. Participants with a history of falls were classified as fallers, while those with no history of falls were classified as non-fallers. Multiple falls were defined as two or more falls in the past year. Fall-related fractures were defined as fractures of bones in any part of the body due to falls in the past year.

### Statistical analysis

The demographic characteristics of the participants were analyzed using a t-test for continuous variables and the chi-square test for categorical variables. The results are expressed as mean ± standard deviation (SD) or number and ratio (%) according to the characteristics of the variables. Unadjusted and fully adjusted analyses were performed using logistic regression models to examine odds ratios (ORs). Each model was fully adjusted for multiple interrelations between fall and other potential confounding variables such as age, depression, osteoarthritis, osteoporosis, diabetes mellitus, location of residence, family group, vitamin D level, number of medications, Mini-Mental Status Examination in the Korean version of the CERAD assessment packet (MMSE-KC) and BMI. The collected data were analyzed using SPSS 23.0 software (IBM, Inc., Chicago, IL), and a *P*-value of < 0.05 was considered significant.

## Results

The baseline characteristics of the participants are shown in Table 1. Among the 2325 participants, 1111 (47.7%) were males and 1212 (52.3%) were females. A total of 239 (24.1%) females in the faller group had a history of falls in the past year, which was statistically higher than that in males (176, 15.8%). The prevalence of sarcopenia was 18.2% in males, which was statistically higher than that in females (11.8%). The prevalence of severe sarcopenia was not significantly different between the sexes. Sex, age, BMI, HGS, ASMI, and SPPB score were significantly different between the groups. Other socioeconomic characteristics, including years of education, marital status, income, residency, smoking, and chronic comorbidities, were significantly different between the groups.

**Table 1:** Baseline characteristics of the participants according to sex

Characteristic		Male	Female	*P*
		(*n* = 1111)	(*n* = 1212)	
Age (mean ± SD)		76.87 ± 3.8	76.11 ± 3.8	< 0.001*
BMI (mean ± SD)		23.96 ± 2.9	24.8 ± 3.0	< 0.001*
HGS, kg (mean ± SD)		32.23 ± 5.8	21.10 ± 3.9	< 0.001*
ASMI, kg/m^2^ (mean ± SD)		7.04 ± 0.8	5.83 ± 0.7	< 0.001*
SPPB total (mean ± SD)		11.09 ± 1.2	10.59 ± 1.5	< 0.001*
Sarcopenia^a^, n (%)		202(18.2)	143(11.8)	< 0.001*
Severe sarcopenia^b^, n (%)		44(4.0)	39(3.2)	0.335
Fall in past year, n (%)		176(15.8)	293(24.1)	< 0.001*
Two or more falls in the past year, n (%)		27(2.4)	41(3.4)	0.178
Fall-related fracture, n (%)		21(1.9)	43(3.5)	0.015*
Medications, n (mean ± SD)		3.64 ± 3.0	3.30 ± 2.6	***0.005***
Years of education, n (%)	< 6	298 (26.0)	688 (56.8)	< 0.001*
	7–12	473 (42.6)	402 (33.2)	
	> 13	349 (31.4)	122 (10.1)	
Marital status, n (%)	Married	1021 (91.9)	788 (65.0)	< 0.001*
Income, million won per month, n (%)^c^	> 3	216 (19.4)	225 (18.6)	< 0.001*
	1–3	553 (49.8)	447 (36.9)	
	< 1	342 (30.8)	540 (44.6)	
Residency, n (%)	Urban	867 (77.9)	1029 (85.1)	< 0.001*
	Rural	244 (22.1)	183 (14.9)	
Current smoker, n (%)		730 (65.7)	22 (1.8)	< 0.001*
Alcohol use, n (%)		661 (59.5)	709 (58.0)	0.46
Hypertension, n (%)		595 (53.8)	714 (58.9)	0.19
Dyslipidemia, n (%)		277 (24.9)	497 (41.0)	< 0.001*
Diabetes mellitus, n (%)		274 (24.7)	243 (20.0)	0.16
Depression, n (%)		21 (1.9)	44 (3.6)	0.11
Osteoarthritis, n (%)		121 (10.9)	392 (32.3)	< 0.001*
Osteoporosis, n (%)		34 (3.1)	312 (90.2)	< 0.001*
WBC, 10^9^/L (mean ± SD)		5.87 ± 1.5	5.77 ± 1.6	< 0.001*
Hb, mmol/L (mean ± SD)		14.11 ± 1.3	12.87 ± 1.1	< 0.001*
25(OH) D (ng/ml, SD)		24.83 ± 9.3	22.63 ± 10.6	< 0.001*
MMSE-KC (mean ± SD)		26.28 ± 2.8	25.32 ± 3.3	< 0.001*

Abbreviations: *BMI* body mass index; *HGS* handgrip strength; *ASMI* appendicular skeletal muscle mass index; *SPPB* short physical performance battery; *WBC* white blood cell; *Hb* hemoglobin; *25(OH) D* 25-hydroxyvitamin D; *MMSE-KC* Mini-Mental Status Examination in the Korean version of the CERAD assessment packet

^a^ Sarcopenia: Low ASMI (< 7.0 kg/m^2^ for males and < 5.4 kg/m^2^ for females) and either a low HGS (< 28 kg for males and < 18 kg for females) or low physical performance (SPPB score ≤ 9 for both sexes)

^b^Severe sarcopenia: Low ASMI with a low HGS and low physical performance

^c^1 million Korean won = USD 900

*P < 0.05

Table 2 shows a comparison of the non-faller and faller groups according to the variables of the sarcopenia definition, physical performance parameters, and sex. Among males, the faller group had lower HGS, and SPPB score and a higher proportion of patients with severe sarcopenia than the non-faller group. Among females, the faller group had lower HGS, ASMI, and SPPB scores than the non-faller group. In females, sarcopenia was more prevalent in the faller group than the non-faller group; however, no significant difference was observed in those with severe sarcopenia.

**Table 2:** Sarcopenia parameters of the non-faller group and faller group according to sex

Characteristic	Male		*P*	Female		*P*
	Non-faller (*n* = 935)	Faller (*n* = 176)		Non-faller (*n* = 919)	Faller (*n* = 293)	
HGS, kg (mean ± SD)	32.6 ± 5.8	30.5 ± 5.8	< 0.001*	21.3 ± 3.9	20.7 ± 3.8	0.044*
ASMI, kg/m^2^ (mean ± SD)	7.04 ± 0.8	6.97 ± 0.8	0.297	5.86 ± 0.7	5.74 ± 0.7	0.014*
SPPB (mean ± SD)	11.2 ± 1.2	10.6 ± 1.6	< 0.001*	10.6 ± 1.4	10.3 ± 1.8	0.013*
Sarcopenia^a^ (n, %)	162 (17.3)	40 (22.7)	0.109	98 (10.7)	45 (15.4)	0.037*
severe Sarcopenia^b^ (n, %)	29 (3.1)	7 (8.5)	0.001*	32 (3.5)	7 (2.4)	0.356

Abbreviations: *HGS* hand grip strength; *ASMI* appendicular skeletal muscle mass index; *SPPB* short physical performance battery

^a^ Sarcopenia: Low ASMI (< 7.0 kg/m^2^ for males and < 5.4 kg/m^2^ for females) and either a low HGS (< 28 kg for males and < 18 kg for females) or low physical performance (SPPB score ≤ 9 for both sexes)

^b^Severe sarcopenia: Low ASMI with a low HGS and low physical performance

*P < 0.05

Table 3 shows the unadjusted and fully adjusted logistic regression analysis results of sarcopenia definition and other parameters defined by the AWGS according to a history of fall. The male faller group had a significantly lower HGS (OR = 1.670, 95% confidence interval [CI] = 1.171–2.382), and a lower SPPB score (OR = 2.439, 95% CI = 1.600–3.718) in the unadjusted model. In contrast, the female faller group had a significantly lower ASMI (OR = 1.392, 95% CI = 1.048–1.850), lower SPPB score (OR = 1.505, 95% CI = 1.110–2.042), and higher prevalence of sarcopenia (OR = 1.520, 95% CI = 1.039–2.224) in the unadjusted model. Meanwhile, the male faller group had a significantly lower HGS (OR = 1.508, 95% CI = 1.028–2.211), lower SPPB score (OR = 2.068, 95% CI = 1.308–3.271), and higher prevalence of severe sarcopenia (OR = 2.417, 95% CI = 1.224–4.771) in the fully adjusted model. However, the female faller group only had a low ASMI in the fully adjusted model (OR = 1.419, 95% CI = 1.058–1.903). Other physical performance parameters, including lower SPPB score and sarcopenia, were attenuated when the confounding factors were considered.

**Table 3:** Logistic regression analysis of sarcopenia parameters and fall history according to sex

Characteristic	Unadjusted model		Fully adjusted model	
	Male	Female	Male	Female
	OR (95% CI)	OR (95% CI)	OR (95% CI)	OR (95% CI)
Low HGS^a^	1.670 (1.171–2.382)*	1.109 (0.803–1.531)	1.508 (1.028–2.211)*	0.941 (0.669–1.324)
Low ASMI^a^	0.845 (0.611–1.167)	1.392 (1.048–1.850)*	0.828 (0.593–1.157)	1.474 (1.077–2.016)*
Low SPPB^a^	2.439 (1.600–3.718)*	1.505 (1.110–2.042)*	2.159 (1.365–3.414)*	1.269 (0.909–1.772)
Sarcopenia ^b^	1.403 (0.949–2.075)	1.520 (1.039–2.224)*	1.292 (0.834–2.003)	1.191 (0.973–1.973)
Severe sarcopenia^c^	2.911 (1.526–5.550)*	0.678 (0.296–1.554)	2.538 (1.274–5.056)*	0.542 (0.231–1.272)

Abbreviations: *OR* odds ratio; *CI* confidence interval; *HGS* hand grip strength; *ASMI* appendicular skeletal muscle mass index; *SPPB* short physical performance battery

The fully adjusted model was adjusted for age, depression, osteoarthritis, osteoporosis, diabetes mellitus, location of residence, family group, vitamin D level, number of medications, MMSE-KC score, and body mass index

^a^ Low HGS, < 28 kg for males and < 18 kg for females; low ASMI, < 7.0 kg/m^2^ for males and < 5.4 kg/m^2^ for females; low SPPB, score ≤ 9 for both sexes

^b^ Sarcopenia: Low ASMI (< 7.0 kg/m^2^ for males and < 5.4 kg/m^2^ for females) and either a low HGS (< 28 kg for males and < 18 kg for females) or low physical performance (SPPB score ≤ 9 for both sexes)

^c^Severe sarcopenia: Low ASMI with a low HGS and low physical performance

*P < 0.05

Table 4 shows the logistic regression analysis results of sarcopenia parameters for fall-related fractures according to sex. In the unadjusted model, the male fall-related fracture group had a significantly lower HGS (OR = 2.573, 95% CI = 1.072–6.178), and SPPB score (OR = 4.928, 95% CI = 2.002–12.131), and a higher prevalence of sarcopenia (OR = 2.842, 95% CI = 1.162–6.951) and severe sarcopenia (OR = 4.264, 95% CI = 1.208–14.055). Similarly, in the unadjusted model, the female fall-related fracture group had a significantly lower SPPB score (OR = 2.548, 95% CI = 1.380–4.704) and a higher prevalence of sarcopenia (OR = 2.36, 95% CI = 1.137–4.899). In the fully adjusted model, both male and female fall-related fracture groups had a significantly lower SPPB (OR = 4.167, 95% CI = 1.449–11.986, and OR = 2.607, 95% CI = 1.315–5.171, respectively). However, in the fully adjusted model, a high prevalence of sarcopenia was found only in the female fall-related fracture group (OR = 2.497, 95% CI = 1.142–5.434).

**Table 4:** Logistic regression analysis of sarcopenia parameters for fall-related fractures according to sex

Characteristic	Unadjusted model		Fully adjusted model	
	Male	Female	Male	Female
	OR (95% CI)	OR (95% CI)	OR (95% CI)	OR (95% CI)
Low HGS^a^	2.573 (1.072–6.178)*	1.133 (0.553–2.321)	2.169 (0.821–5.729)	0.997 (0.461–2.155)
Low ASMI^a^	0.795 (0.332–1.903)	1.600 (0.864–2.963)	0.459 (0.146–1.261)	1.322 (0.656–2.663)
Low SPPB^a^	4.928 (2.002–12.131)**	2.548 (1.380–4.704)*	4.167 (1.449–11.986)*	2.607 (1.315–5.171)*
Sarcopenia ^b^	2.842 (1.162–6.951)*	2.36 (1.137–4.899)*	1.891 (0.658–5.433)	2.497 (1.142–5.434)*
Severe sarcopenia^c^	4.264 (1.208–14.055)*	1.420 (0.332–6.087)	2.965 (0.725–12.116)	1.494 (0.325–6.869)

Abbreviations: *OR* odds ratio; *CI* confidence interval; *HGS* hand grip strength; *ASMI* appendicular skeletal muscle mass index; *SPPB* short physical performance battery

The fully adjusted model was adjusted for age, depression, osteoarthritis, osteoporosis, diabetes mellitus, location of residence, family group, vitamin D level, number of medications, MMSE-KC score, and body mass index

^a^ Low HGS, < 28 kg for males and < 18 kg for females; low ASMI, < 7.0 kg/m^2^ for males and < 5.4 kg/m^2^ for females; low SPPB, score ≤ 9 for both sexes

^b^ Sarcopenia: Low ASMI (< 7.0 kg/m^2^ for males and < 5.4 kg/m^2^ for females) and either a low HGS (< 28 kg for males and < 18 kg for females) or low physical performance (SPPB score ≤ 9 for both sexes)

^c^Severe sarcopenia: Low ASMI with a low HGS and low physical performance

*P < 0.05

**P < 0.01

Table 5 shows logistic regression analysis results of sarcopenia parameters for multiple falls, defined as ≥2 falls in the previous year, according to sex. In males, in the unadjusted model, a lower HGS (OR = 4.396, 95% CI = 2.030–9.518), and SPPB score (OR = 2.760, 95% CI = 1.143–6.661), and sarcopenia (OR = 2.308, 95% CI = 1.022–5.216) were statistically significant factors for multiple falls. However, in males, in the fully adjusted model, only a low HGS showed a strong association with multiple falls (OR = 3.980, 95% CI = 1.706–9.284, *P* < 0.001).

**Table 5:** Logistic regression analysis of sarcopenia parameters for multiple falls according to sex

Characteristic	Unadjusted model		Fully adjusted model	
	Male	Female	Male	Female
	OR (95% CI)	OR (95% CI)	OR (95% CI)	OR (95% CI)
Low HGS^a^	4.396 (2.030–9.518)**	1.329 (0.641–2.757)	3.980 (1.706–9.284)**	1.174 (0.558–2.469)
Low ASMI^a^	0.727 (0.334–1.580)	0.858 (0.415–1.774)	0.678 (0.271–1.700)	0.908 (0.422–1.950)
Low SPPB^a^	2.760 (1.143–6.661)*	1.597 (0.800–3.186)	1.866 (0.700–4.973)	1.357 (0.645–2.858)
Sarcopenia ^b^	2.308 (1.022–5.216)*	1.070 (0.412–2.778)	2.077 (0.808–5.334)	1.032 (0.399–2.666)
Severe sarcopenia^c^	3.180 (0.920–10.99)	0.745 (0.100–5.566)	2.573 (0.666–9.934)	0.530 (0.068–4.130)

Abbreviations: *OR* odds ratio; *CI* confidence interval; *HGS* hand grip strength; *ASMI* appendicular skeletal muscle mass index; *SPPB* short physical performance battery

The fully adjusted model was adjusted for age, depression, osteoarthritis, osteoporosis, diabetes mellitus, location of residence, family group, vitamin D level, number of medications, MMSE-KC score, and body mass index

^a^ Low HGS, < 28 kg for males and < 18 kg for females; low ASMI, < 7.0 kg/m^2^ for males and < 5.4 kg/m^2^ for females; low SPPB, score ≤ 9 for both sexes

^b^ Sarcopenia: Low ASMI (< 7.0 kg/m^2^ for males and < 5.4 kg/m^2^ for females) and either a low HGS (< 28 kg for males and < 18 kg for females) or low physical performance (SPPB score ≤ 9 for both sexes)

^c^Severe sarcopenia: Low ASMI with a low HGS and low physical performance

*P < 0.05

**P < 0.01

## Discussion

We investigated the associations between sarcopenia parameters and the incidence of falls and fall-related fractures according to sex. Female sex is a known risk factor for falls, and in this study, the impact of sarcopenia on falls and fall-related fractures differed between the sexes [5, 16].

In males, low HGS (which represents muscle strength) and low physical performance parameters such as low SPPB score had an impact on a history of falls; in females, only a low ASMI (which indicates muscle mass) had an effect. The definition of sarcopenia according to the AWGS, which is characterized by low muscle mass plus low muscle strength or low physical performance, did not show a significant relationship with a history of falls in either sex group. Severe sarcopenia, defined as sarcopenia plus low physical performance, showed a significant relationship with a history of falls. These results can be explained by the different risk of falls in males and females.

The risk factors for sarcopenia are similar to those for age-related conditions [20]. Since the risk factors for falls are associated with those for sarcopenia, the correlation between falls and sarcopenia has been studied. However, the association between sarcopenia and falls is controversial. Several previous studies have evaluated the association between sarcopenia, defined by EWGSOP diagnostic criteria, and falls. In an Italian study, Landi et al. studied the relationship between sarcopenia and the 2-year risk of falls. In a total of 250 patients aged ≥80 years, falls were highly prevalent among older persons with sarcopenia regardless of sex differences (OR = 3.23, 95% CI = 1.25–8.29) [21]. In another study in Chile conducted in 1006 community-dwelling participants aged > 60 years, falls were associated with sarcopenia defined by the EWGSOP [22]. However, other studies have reported a non-significant association between sarcopenia and falls. In a Columbian study conducted in 534 participants (mean age = 74.4, SD = 8.2), falls that occurred in the previous year showed no significant relationship with sarcopenia [23]. In a previous UK study conducted in 286 participants with a mean age of 76.1 years, no significant associations were observed between sarcopenia and a history of falls [24]. Previous studies mainly defined sarcopenia based on the EWGSOP criteria; however, due to the differences in body composition between Asians and Caucasians, sarcopenia defined by the AWGS was found to be more relevant in Asians. Even with the same BMI, Asians have a higher body fat percentage, lower skeletal muscle mass, and prominent abdominal obesity than Caucasians [25]. Only one study on sarcopenia, defined by the AWGS, and falls was examined. A 2-year prospective Japanese study conducted in 162 individuals showed a significant risk of falls in the sarcopenia group in the adjusted logistic regression analysis (OR = 7.68, 95% CI = 1.41–41.77) [26]. However, this study had certain limitations—it had a small sample size, 61 of the 223 participants withdrew from the study, and the study was conducted in rural areas with a limited population.

In this study, no correlation was found between falls and sarcopenia, but a relationship was found between falls and the sarcopenia component parameters. In males, a correlation was found between low muscle strength, low physical performance, and a history of falls. These results were similar to those reported a previous study. In a recent study conducted in Taiwan, low HGS was associated with a history of falls in both sexes [27]. Our study also showed the same results when the average HGS of fallers was compared to thar of non-fallers. However, when the cutoff value of the AWGS was set, low HGS increased the incidence of falls in males, but not in females. Although a mean difference in muscle strength was observed between the faller group and non-faller group, only males showed a significant difference when the muscle strength decreased, which was one of the criteria for diagnosing sarcopenia. Additionally, this study showed that, in males, a low HGS had a strong association with multiple falls, which was consistent with the results of previous studies [28]. In both sexes, the faller group showed a lower mean SPPB value, which indicates lower physical function; this finding is consistent with that reported by previous studies [29]. When compared by AWGS criteria, it is still correlated in the unadjusted logistic regression analysis. However, in females, attenuation was observed in the fully adjusted regression analysis. Low physical function is thought to be an important risk factor for falls in males; in females, other covariates have a greater effect on a history of falls.

In this study, the faller and non-faller groups showed sex differences in terms of muscle mass. In males, a significant difference was observed in ASMI between the faller group and non-faller group, but a lower ASMI was observed in the female faller group. Moreover, a significant association was observed in females in the unadjusted and fully adjusted logistic regression analyses. This is thought to be due to the sex differences in body composition [30]. Females are more strongly affected by alterations in body composition as they have a high body fat percentage and low muscle mass, which leads to the differences in physical performance and balance [31]. In a previous study, Walters et al. reported that a low ASMI was associated with balance deficit and fall incidence in females, but no association was found in males [32]. This finding is consistent with the results of our study, and the sex-related differences in body composition affect balance and the risk of falls. This difference between males and females can be explained by the difference in body composition and is more strongly associated with the lack of balance due to low physical performance in males. In contrast, the fat mass and muscle mass in females are more strongly associated with a lack of balance.

This study has several limitations. This study was cross-sectional in nature. However, this study is considered significant as it is the first large cohort study with > 2000 participants to examine the relationship between sarcopenia, defined by the AWGS, and falls. Further prospective studies and randomized controlled studies are warranted to confirm our findings. Second, we used a self-reported questionnaire, which might have led to a possible recall bias of retrospective individual memories. If falls and fall-related fractures occur during old age, it is considered as a serious event; hence, details of the history of falls and falls number might be accurately remembered. However, the falls possibly occurred 1 year or more before. Third, we did not measure physical activity level as a covariable factor, which is a risk factor for both falls and sarcopenia [33, 34]. Finally, since this study was conducted in older adults, which is one of the sarcopenia component parameters, the results of the physical performance tests may have varied depending on the patients’ fatigue status or body condition as a result of fall or fall-related fracture history. Hence, our results may not be considered generalizable. This problem has also been addressed in other geriatric studies.

## Conclusion

This is the first large cross-sectional cohort study to examine sarcopenia, defined by the AWGS, and its correlation with falls in older Korean population. Our results showed that the incidence of falls did not increase in the group with sarcopenia defined by the AWGS. In this study, we found that falls and fall-related fractures were higher in female than in male patients, and sex differences in each sarcopenia component parameter affected the history of falls. Among sarcopenia components, low muscle strength, low physical performance in males, and low muscle mass in females were significant risk factors. Therefore, when studying fall risk in old age, sex differences in body composition and physical function should be considered.

## Data Availability

All the cohort data that support the findings of this study are available from the KFACS and open to all researchers on reasonable research requests. All published articles and news articles using the KFACS database, data provision manuals, and contact information are available on the KFACS website (http://www.kfacs.kr).

## Abbreviations

KFACS: Korean Frailty and Aging Cohort study;
AWGS: Asian Working Group for Sarcopenia
SPPB: Short physical performance battery
HGS: Hand grip strength
ASMI: Appendicular skeletal muscle mass index
BMI: Body mass index
EWGSOP: European Working Group on Sarcopenia in Older People
OR: Odds ratio
CI: Confidence interval

## References

[1] Tinetti ME (2003). Preventing falls in elderly persons. N Engl J Med.

[2] Berry SD, Miller RR (2008). Falls: epidemiology, pathophysiology, and relationship to fracture. Curr Osteoporosis Rep.

[3] Pfortmueller C, Lindner G, Exadaktylos A (2014). Reducing fall risk in the elderly: risk factors and fall prevention, a systematic review. Minerva Med.

[4] Welmer A-K, Rizzuto D, Laukka EJ, Johnell K, Fratiglioni L (2017). Cognitive and physical function in relation to the risk of injurious falls in older adults: a population-based study. Journals Gerontology: Series A.

[5] Gale CR, Cooper C, Aihie Sayer A (2016). Prevalence and risk factors for falls in older men and women: the English longitudinal study of ageing. Age Ageing.

[6] O'Loughlin JL, Robitaille Y, Boivin J-F, Suissa S (1993). Incidence of and risk factors for falls and injurious falls among the community-dwelling elderly. Am J Epidemiol.

[7] Dionyssiotis Y (2012). Analyzing the problem of falls among older people. Int J General Med.

[8] Walston JD (2012). Sarcopenia in older adults. Curr Opin Rheumatol.

[9] Santilli V, Bernetti A, Mangone M, Paoloni M (2014). Clinical definition of sarcopenia. Clin Cases Mineral Bone Metab.

[10] Volpi E, Nazemi R, Fujita S (2004). Muscle tissue changes with aging. Current Opinion in Clinical Nutrition and Metabolic care.

[11] Shafiee G, Keshtkar A, Soltani A, Ahadi Z, Larijani B, Heshmat R (2017). Prevalence of sarcopenia in the world: a systematic review and meta-analysis of general population studies. J Diabetes Metabolic Disorders.

[12] Clark BC (2019). Neuromuscular changes with aging and sarcopenia. J Frailty Aging.

[13] Clark DJ, Fielding RA (2012). Neuromuscular contributions to age-related weakness. J Gerontology Series A: Biomedical Sciences and Medical Sciences.

[14] Liguori I, Russo G, Aran L, Bulli G, Curcio F, Della-Morte D, Gargiulo G, Testa G, Cacciatore F, Bonaduce D (2018). Sarcopenia: assessment of disease burden and strategies to improve outcomes. Clin Interv Aging.

[15] Al-Aama T (2011). Falls in the elderly: spectrum and prevention. Can Fam Physician.

[16] Du Y, Wang X, Xie H, Zheng S, Wu X, Zhu X, Zhang X, Xue S, Li H, Hong W (2019). Sex differences in the prevalence and adverse outcomes of sarcopenia and sarcopenic obesity in community dwelling elderly in East China using the AWGS criteria. BMC Endocr Disord.

[17] Öztürk ZA, Türkbeyler İH, Abiyev A, Kul S, Edizer B, Yakaryılmaz FD, Soylu G (2018). Health-related quality of life and fall risk associated with age-related body composition changes; sarcopenia, obesity and sarcopenic obesity. Intern Med J.

[18] Chen L-K, Woo J, Assantachai P, Auyeung T-W, Chou M-Y, Iijima K, Jang HC, Kang L, Kim M, Kim S (2020). Asian Working Group for Sarcopenia: 2019 consensus update on sarcopenia diagnosis and treatment. J American Medical Directors Association.

[19] Hilal S, Perna S, Gasparri C, Alalwan TA, Vecchio V, Fossari F, Peroni G, Riva A, Petrangolini G, Rondanelli M (2020). Comparison between appendicular skeletal muscle index DXA defined by EWGSOP1 and 2 versus BIA Tengvall criteria among older people admitted to the post-acute geriatric care unit in Italy. Nutrients.

[20] Grundstrom AC, Guse CE, Layde PM (2012). Risk factors for falls and fall-related injuries in adults 85 years of age and older. Arch Gerontol Geriatr.

[21] Landi F, Liperoti R, Russo A, Giovannini S, Tosato M, Capoluongo E, Bernabei R, Onder G (2012). Sarcopenia as a risk factor for falls in elderly individuals: results from the ilSIRENTE study. Clin Nutr.

[22] Lera L, Albala C, Sánchez H, Angel B, Hormazabal M, Márquez C, Arroyo P (2017). Prevalence of sarcopenia in community-dwelling Chilean elders according to an adapted version of the European working group on sarcopenia in older people (EWGSOP) criteria. J Frailty Aging.

[23] Benjumea A-M, Curcio C-L, Duque G, Gomez F (2018). Dynapenia and sarcopenia as a risk factor for disability in a falls and fractures clinic in older persons. Open access Macedonian journal of medical sciences.

[24] Clynes M, Edwards M, Buehring B, Dennison E, Binkley N, Cooper C (2015). Definitions of sarcopenia: associations with previous falls and fracture in a population sample. Calcif Tissue Int.

[25] Wulan S, Westerterp K, Plasqui G (2010). Ethnic differences in body composition and the associated metabolic profile: a comparative study between Asians and Caucasians. Maturitas.

[26] Matsumoto H, Tanimura C, Tanishima S, Osaki M, Noma H, Hagino H (2017). Sarcopenia is a risk factor for falling in independently living Japanese older adults: a 2-year prospective cohort study of the GAINA study. Geriatr Gerontol Int.

[27] Yang N-P, Hsu N-W, Lin C-H, Chen H-C, Tsao H-M, Lo S-S, Chou P (2018). Relationship between muscle strength and fall episodes among the elderly: the Yilan study, Taiwan. BMC Geriatr.

[28] Cöster ME, Karlsson M, Ohlsson C, Mellström D, Lorentzon M, Ribom E, Rosengren B (2020). Physical function tests predict incident falls: a prospective study of 2969 men in the Swedish osteoporotic fractures in men study. Scandinavian J Public Health.

[29] Veronese N, Bolzetta F, Toffanello ED, Zambon S, De Rui M, Perissinotto E, Coin A, Corti M-C, Baggio G, Crepaldi G (2014). Association between short physical performance battery and falls in older people: the Progetto Veneto Anziani study. Rejuvenation Res.

[30] Wells JC (2007). Sexual dimorphism of body composition. Best Pract Res Clin Endocrinol Metab.

[31] Valentine RJ, Misic MM, Rosengren KS, Woods JA, Evans EM (2009). Sex impacts the relation between body composition and physical function in older adults. Menopause (New York, NY).

[32] Waters DL, Qualls C, Cesari M, Rolland Y, Vlietstra L, Vellas B (2019). Relationship of incident falls with balance deficits and body composition in male and female community-dwelling elders. J Nutr Health Aging.

[33] Freiberger E, Sieber C, Pfeifer K (2011). Physical activity, exercise, and sarcopenia–future challenges. Wien Med Wochenschr.

[34] Low ST, Balaraman T (2017). Physical activity level and fall risk among community-dwelling older adults. J Phys Ther Sci.

